# Human alveolar macrophage response to Mycobacterium tuberculosis: immune characteristics underlying large inter-individual variability

**DOI:** 10.21203/rs.3.rs-2986649/v1

**Published:** 2023-06-08

**Authors:** Wolfgang Sadee, Ian H. Cheeseman, Audrey Papp, Maciej Pietrzak, Michal Seweryn, Xiaofei Zhou, Shili Lin, Amanda M. Williams, Mark D. Wewers, Heather M. Curry, Hao Zhang, Hong Cai, Carine Kunsevi-Kilola, Happy Tshivhula, Gerhard Walzl, Blanca I. Restrepo, Léanie Kleynhans, Katharina Ronacher, Yufeng Wang, Eusondia Arnett, Abul K. Azad, Larry S. Schlesinger

**Affiliations:** The Ohio State University; Texas Biomedical Research Institute; The Ohio State University; The Ohio State University; University of Łódź; The Ohio State University; The Ohio State University; The Ohio State University; The Ohio State University; QED Biosciences, Inc; University of Texas at San Antonio; University of Texas at San Antonio; Stellenbosch University; Stellenbosch University; Stellenbosch University; University of Texas Rio Grande Valley, South Texas Diabetes and Obesity Institute; Stellenbosch University; Stellenbosch University; University of Texas at San Antonio; Texas Biomedical Research Institute; Texas Biomedical Research Institute; Texas Biomedical Research Institute

**Keywords:** Tuberculosis, Alveolar Macrophages, Genomics, Systems Biology

## Abstract

**Background:**

*Mycobacterium tuberculosis* (*M.tb)*, the causative bacterium of tuberculosis (TB), establishes residence and grows in human alveolar macrophages (AMs). Inter-individual variation in *M.tb*-human AM interactions can indicate TB risk and the efficacy of therapies and vaccines; however, we currently lack an understanding of the gene and protein expression programs that dictate this variation in the lungs.

**Results:**

Herein, we systematically analyze interactions of a virulent *M.tb* strain H_37_R_v_ with freshly isolated human AMs from 28 healthy adult donors, measuring host RNA expression and secreted candidate proteins associated with TB pathogenesis over 72h. A large set of genes possessing highly variable inter-individual expression levels are differentially expressed in response to *M.tb* infection. Eigengene modules link *M.tb* growth rate with host transcriptional and protein profiles at 24 and 72h. Systems analysis of differential RNA and protein expression identifies a robust network with *IL1B*, *STAT1*, and *IDO1* as hub genes associated with *M.tb* growth. RNA time profiles document stimulation towards an M1-type macrophage gene expression followed by emergence of an M2-type profile. Finally, we replicate these results in a cohort from a TB-endemic region, finding a substantial portion of significant differentially expressed genes overlapping between studies.

**Conclusions:**

We observe large inter-individual differences in bacterial uptake and growth, with tenfold variation in *M.tb* load by 72h.The fine-scale resolution of this work enables the identification of genes and gene networks associated with early *M.tb* growth dynamics in defined donor clusters, an important step in developing potential biological indicators of individual susceptibility to *M.tb* infection and response to therapies.

## Background

*Mycobacterium tuberculosis* (*M.tb)* infects approximately one-quarter of the world’s population, and nearly 1.3 million people die annually from tuberculosis (TB), a continuing worldwide public health problem (1). Since only a fraction of infected individuals progress to active TB, we must understand the immunopathogenesis of *M.tb* infection, including host susceptibility or resistance factors, to develop effective diagnostic, therapeutic and vaccine strategies tailored to different individuals and populations (2).

Aerosolized *M.tb* enters the alveolar lung space where it is phagocytosed by alveolar macrophages (AMs), unique resident cells with a complex immunologic profile, to become an intracellular pathogen. *M.tb* infection activates several macrophage immunobiological pathways involved in phagocytosis, vesicle trafficking, and triggering of inflammatory cytokines, oxidants, and cell death pathways – all processes of innate immunity. Yet, critical factors in the human host that dictate *M.tb* infection and progression to TB remain uncertain - a roadblock to understanding an individual’s susceptibility to TB (3–5).

The host response to *M.tb* infection ranges from complete clearance of infection to latent, incipient, subclinical and active TB (6). Therapy and vaccine trials further highlight response variability across populations (2, 7) with BCG vaccination trials attaining only 50% efficacy (8). Evolutionary adaptation of *M.tb* to the host is considered a main factor modulating virulence, host response and TB severity (9, 10). For example, evasion of immune surveillance by suppression of IL1B was proposed to be largely dictated by virulence of the *M.tb* strain (9); however, expression of differentially expressed (DE) genes during infection with a single *M.tb* strain can vary > 10-fold between individuals (11). Therefore, both genetic (12) and environmental/epigenetic (13–16) host factors also play a role, while being less well understood.

Heritability estimates of susceptibility to TB range from 80% (twin and population studies) to 25–50% (polygenic risk scores) (17); yet, GWAS-significant genetic variants alone fail to account for most of the estimated heritability (17, 18). Both *in vitro* and *in vivo* transcriptome studies of *M.tb*-infected macrophages and other immune cells reveal DE candidate genes (19–22), resulting in gene networks (4, 14, 23–25), including type I IFN-associated signatures in active TB (22). Similarly, protein studies invoke factors associated with TB status, including indoleamine oxidase 1 (IDO1), a marker of active TB (26). Complexity of these interacting processes confound predictions of individual TB risk.

Our study addresses a critical gap in the field, the poorly understood substantial inter-person variability in response to a single *M.tb* strain in the lungs. We address this by characterizing immune response genes and pathways that differ between individuals during infection of human AMs from healthy donors. We identify host DE genes and functional networks with highly variable expression between AM donors correlating with *M.tb* growth dynamics in AMs, some already recognized as potential biomarkers of active TB (9, 22, 26–28). The approach builds the framework for understanding an individual’s susceptibility to *M.tb* and sets the stage for future studies designed to understand different responses to distinct *M.tb* strains, vaccines, and therapies.

## Results

### Heterogeneity in M.tb uptake, adaptation and growth in human AMs among donors

**Figure 1:**
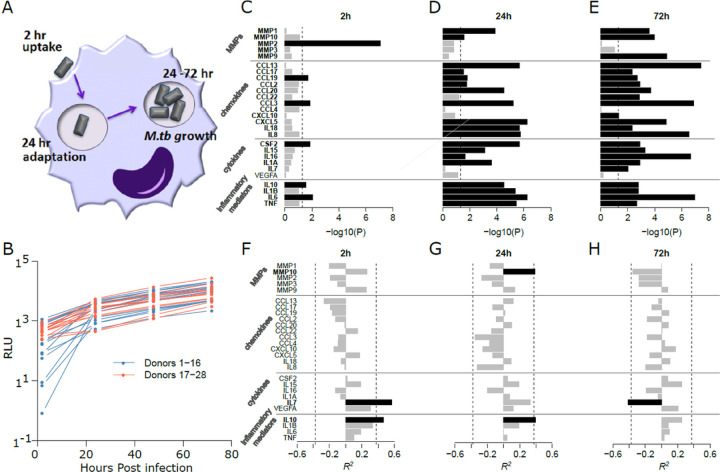
Intracellular *M.tb* growth in human alveolar macrophages (AMs) and its correlation with cellular protein secretion in culture media. **Panel A.** Schematic of *M.tb* cell attachment and uptake, adaptation and growth in human AMs. **Panel B**. Changes in normalized luminescence RLU values over the course of *M.tb* infection. The x-axis shows hours post infection, with RLUs representing mostly uptake and adaptation at 2h and 24h, and growth rates (48h/24h or 72h/48h) (see Additional file 1: Table S1; results shown here are with MOI 2:1). **Panels C, D**, and **E**. Differentially secreted proteins (4 inflammatory mediators, 6 cytokines, 12 chemokines, and 5 MMPs) post infection at 2, 24, and 72h respectively (infected/controls at each time point). The bars represent adjusted p values (-log10), with significant proteins shown with black bars. **Panels F, G**, and **H.** Correlations (R^2^) between protein levels and *M.tb* generation time (time for doubling during 48 to 72h period; i.e. positive correlations suggesting improved *M.tb* growth containment). Positive R^2^ values indicate that increased levels correlate with increased generation times, and *vice versa*.

We isolated human AMs from 28 adult healthy donors (Table 1) and soon after (within 6h) infected them with a virulent *M.tb* strain (H_37_R_v_) engineered to emit light (expressed in relative luminescence unit or RLU values) over 2h, and followed RLUs over 72h (Fig. 1A,B). Given the focus of this study on host variation in response to infection, we selected a single, well-studied strain (H_37_R_v_). The assays presented throughout were conducted at a multiplicity of infection (MOI) of 2:1 (*M.tb*/AM cells), and macrophage monolayers were maintained throughout the assays. RLUs correlate with *M.tb* colony forming units (CFUs), jointly reflecting the number of intracellular bacilli and microbial metabolic activity (29). RLU levels at 2–24h post-infection largely reflect *M.tb* cellular uptake and early intracellular adaptation. After correcting for batch effects, large variations in RLU values were detectable at each time point (**Additional file 1: Table S1**). Since large RLU differences at 2h post infection could have arisen from variations in early adaptation of *M.tb* to the intracellular environment, they were not further considered. At 24h, early events largely stabilized, and RLU increases from 48 to 72h accurately reflected *M.tb* growth rates. AMs with the highest and lowest RLUs displayed a nearly tenfold difference at 72h. RLU ratios (48/24h and 72/48h) were similar using an MOI of 10:1 (**Additional file 1: Table S1**). During exponential growth, the generation time is the time interval over which a bacterial population doubles in number, and represents the growth rate. We estimated the generation time for *M.tb* in each donor from RLU values at 48–72h post infection, finding these to be broadly consistent with prior estimates of ~ 15h while varying two-fold between donors. To identify proteins and genes that influence *M.tb*-AM interactions, we focused on generation times (48–72h) in this study, since RLU levels prior to 48h post infection reflect *M.tb* uptake, adaptation, and early growth combined and, in addition, since some values at the early time points fell below the detection limit of the instrument.

### Human AM-secreted proteins capture the immune response to M.tb infection

To assess the inflammatory mediator response by macrophages to *M.tb* infection, we measured 27 secreted proteins, previously implicated in cellular responses to *M.tb* infection, in all AMs over 72h (**Additional file 2: Table S2**). At 2h, 6 of the 27 proteins (22.2%) were significantly differentially produced after *M.tb* infection (Fig. 1C), increasing to 23 proteins (85.2%) at 72h (Fig. 1E), all with increased secretion in infected cells. Differential secretion varied over time, with IL6 and MMP2 displaying the highest levels at 2h, and IL6, CCL13, and CCL3 at 72h. Pronounced *M*.*tb*-stimulated secretion of MMP2 only at 2h (without stimulated mRNA expression; not shown) suggests a mechanism of rapid release from the cell into the extracellular matrix rather than *de novo* transcription. Inflammatory mediators including IL1β, TNF, and IL6 displayed significant differential secretion across the time points, indicating their contribution to a pro-inflammatory reaction to infection.

We then tested whether protein levels secreted from infected cells correlated with *M.tb* generation times (Fig. 1F–H; **Additional file 3: Table S3**). Three proteins were significantly correlated with *M.tb* generation times (FDR adj. p values < 0.05): IL7 (2h, 72h), IL10 (2h, 24h), and MMP10 (24h), with Pearson correlation reaching R^2^ = 0.5. However, IL7 levels (typically produced by lymphoid cells) were at the lower range of the assay sensitivity and may not be reliable. High IL10 levels significantly correlated with longer generation times (slower growth) (r = 0.46 and 0.49 at 2h and 24h, respectively). MMP10 levels were significantly correlated with slower growth at 24h, though switch to become positively (though non-significantly) correlated with rapid growth at 72h. These results support the notion that protein expression profiles over the first 3 days of infection influence *M.tb*-AM interaction dynamics.

### Transcriptomes of uninfected control and M.tb -infected human AMs reveal differentially expressed gene profiles

Transcript profiles were assessed for each AM sample at 2, 24, and 72h post infection using AmpliSeq (30). This method has been shown to be scalable across a large concentration range (30). At each time point 10,000–14,000 mRNAs were detectable, and reads per million (RPM) from replicate assays were highly correlated (r^2^ ≥ 0.99), enabling sensitive detection of differentially expressed (DE) genes. A principal component analysis (PCA) of all datasets from control and infected AMs revealed that 52% of the variance in gene expression between time points resulted from exposure to *ex vivo* culture conditions alone, while *M.tb*-induced RNA expression changes were smaller but increased over time (**Additional file 4: Supplementary Fig. 1**). We previously showed that *ex vivo* cultured HAMs rapidly (starting at approximately 6h) begin to display the transcriptional profile of monocyte-derived macrophages (MDM) suggesting a de-differentiation process is driving the changes we see (30). The dataset we explore here supports this (**Additional file 5: Supplementary Fig. 2**). Canonical human AM markers are highly expressed at 2h in both control and infected samples, but downregulated over 72h. In contrast, MDM markers are upregulated during *ex vivo* culture.

**Figure 2:**
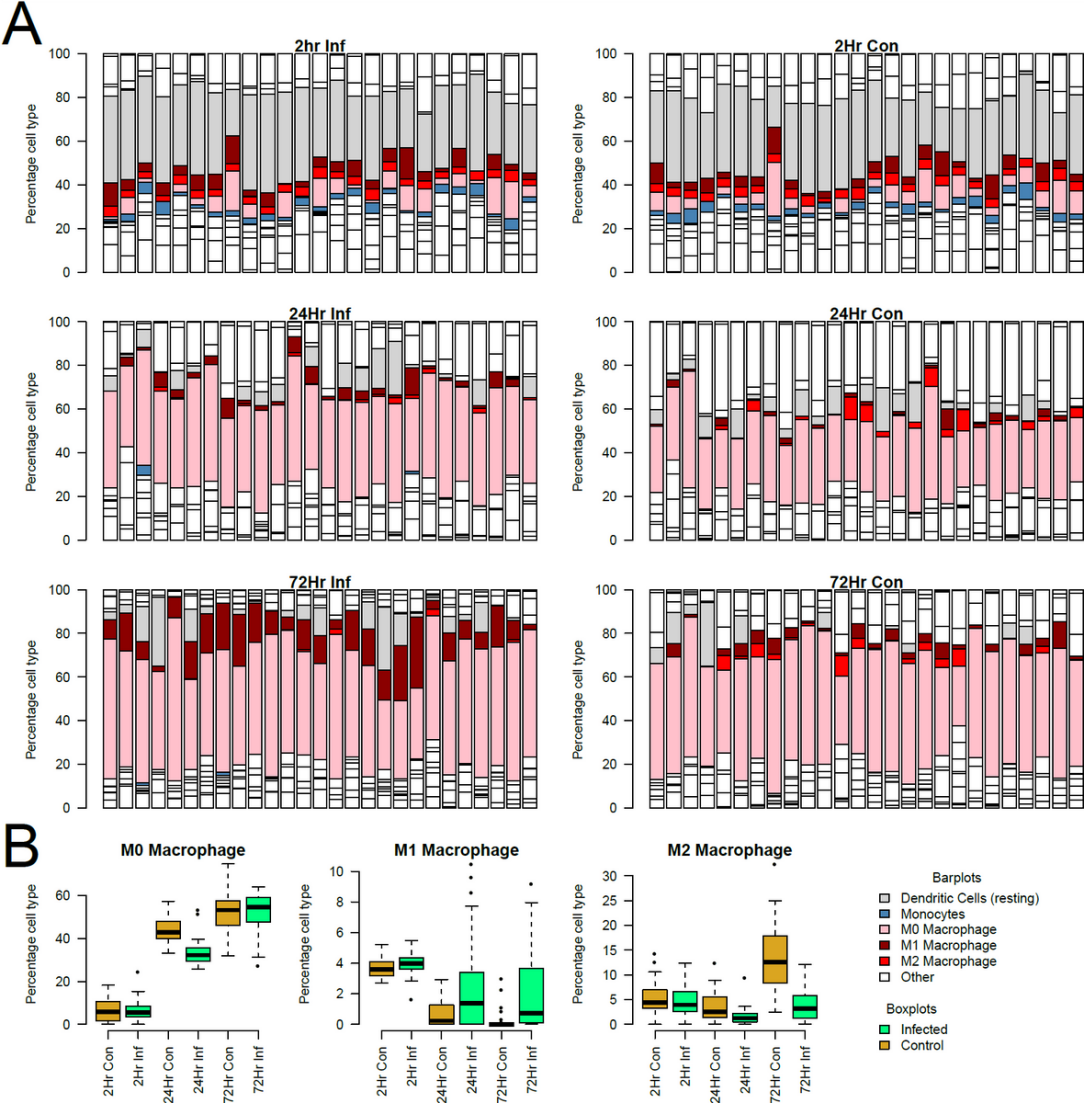
Cell type deconvolution with CIBERSORT-X on AmpliSeq profiles at 2, 24 and 72h in infected and control cells. **Panel A.** Distribution of cell types visualized by the color code shown above. Shown on the left are infected and on the right are control cells at 2, 24 and 72h, respectively, from top to bottom. Note the preponderance of M1 type cells at 24h infected AMs, and of M2 type cells at 72h in controls. **Panel B.** Boxplots showing the proportion of M1 (left) and M2 (right) cells in infected (yellow boxes) and control (green boxes) cells at each time point.

Although there is a strong impact of *ex vivo* culture on gene expression profiles at 24 and 72h, infected samples are clearly distinguished from control samples by PCA of gene expression and by differential expression of secreted proteins. Macrophage polarization is often simplified to the trajectory towards M1-like or M2-like states, the former representing a pro-inflammatory state. To explore the relevance of these polarized states to differences between control and infected samples we first tested whether the phenotype of the cultured macrophages changed over time by deconvolution of the AmpliSeq profiles with CIBERSORT-X. Across both control and infected samples we saw a shift to an M0 macrophage population – likely reflecting the proposed shift of cells towards an MDM-like state. We identified a higher proportion of M1-like macrophages in infected samples than control samples. Conversely, in control AMs a shift toward M2 cell type was observed by 72h (Fig. 2; **Additional file 5: Supplementary Fig. 2**).

**Figure 3:**
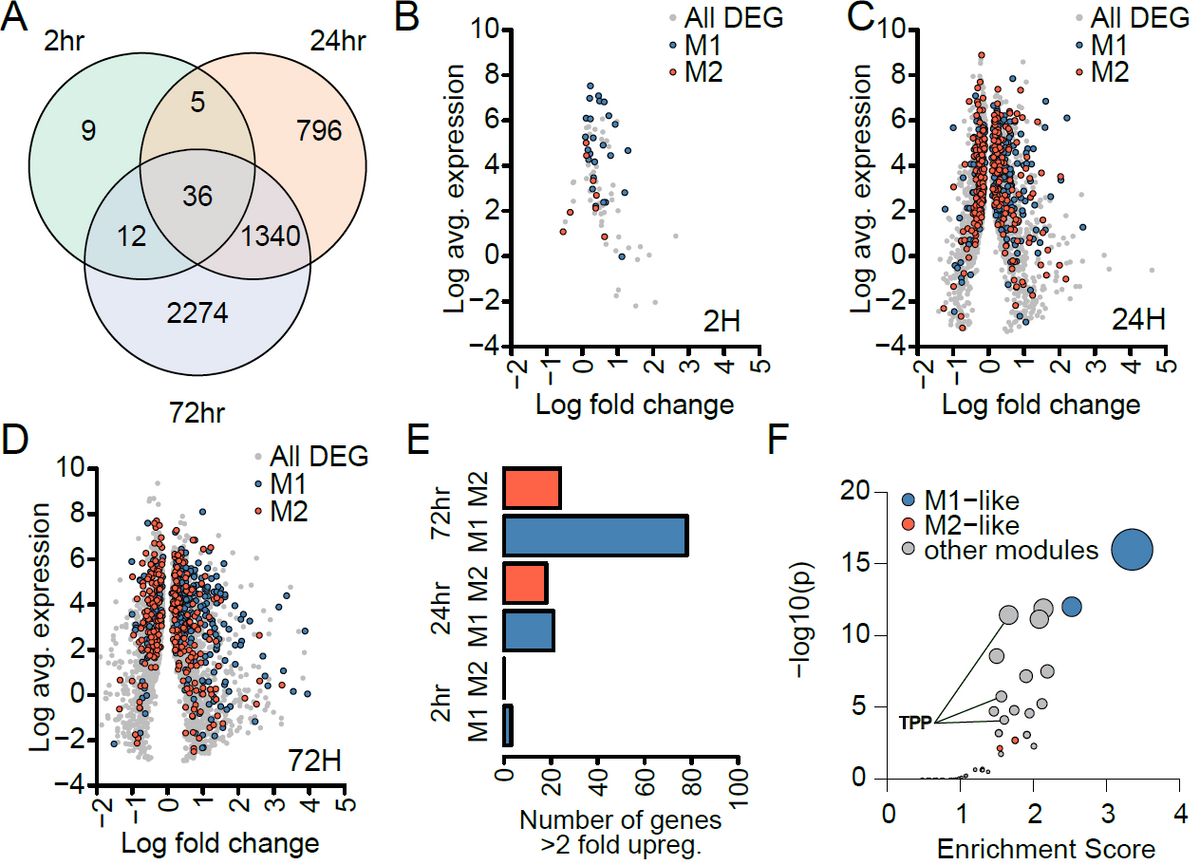
The transcriptional response to *M.tb* infection by 28 human AMs. **Panel A.** Venn diagram of differentially expressed (DE) genes between *M.tb*-treated and controls at three time points. Total number of genes with detectable expression (≥1RPM) was ~12,000. DE genes were detected with DESeq2 (adjusted p values <0.05): 62 at 2h, 2,177 at 24h, and 3,662 at 72h, with overlaps displayed in the Venn diagram. **Panel B-D:** Log_2_ fold change and log_2_ average expression for DE genes at 2h (B), 24h (C) and 72h (D). M1 and M2 genes are shown in blue and red respectively. **Panel E.** The number of DE genes with >2-fold upregulation defined as ‘M1-like’ or ‘M2-like’. This uses an independent definition of ‘M1-like’ and ‘M2-like’ genes to (F). **Panel F.** Gene set enrichment analysis of DE genes from 72h using 48 gene expression modules previously determined as operational in macrophage differentiation. Modules annotated as ‘M1-like’ or ‘M2-like’ are shown in blue and red, another major grouping of modules was related to *ex vivo* TPP (TNF, PGE2 and P3C) stimulation in (33) and are highlighted by a line.

**Figure 4:**
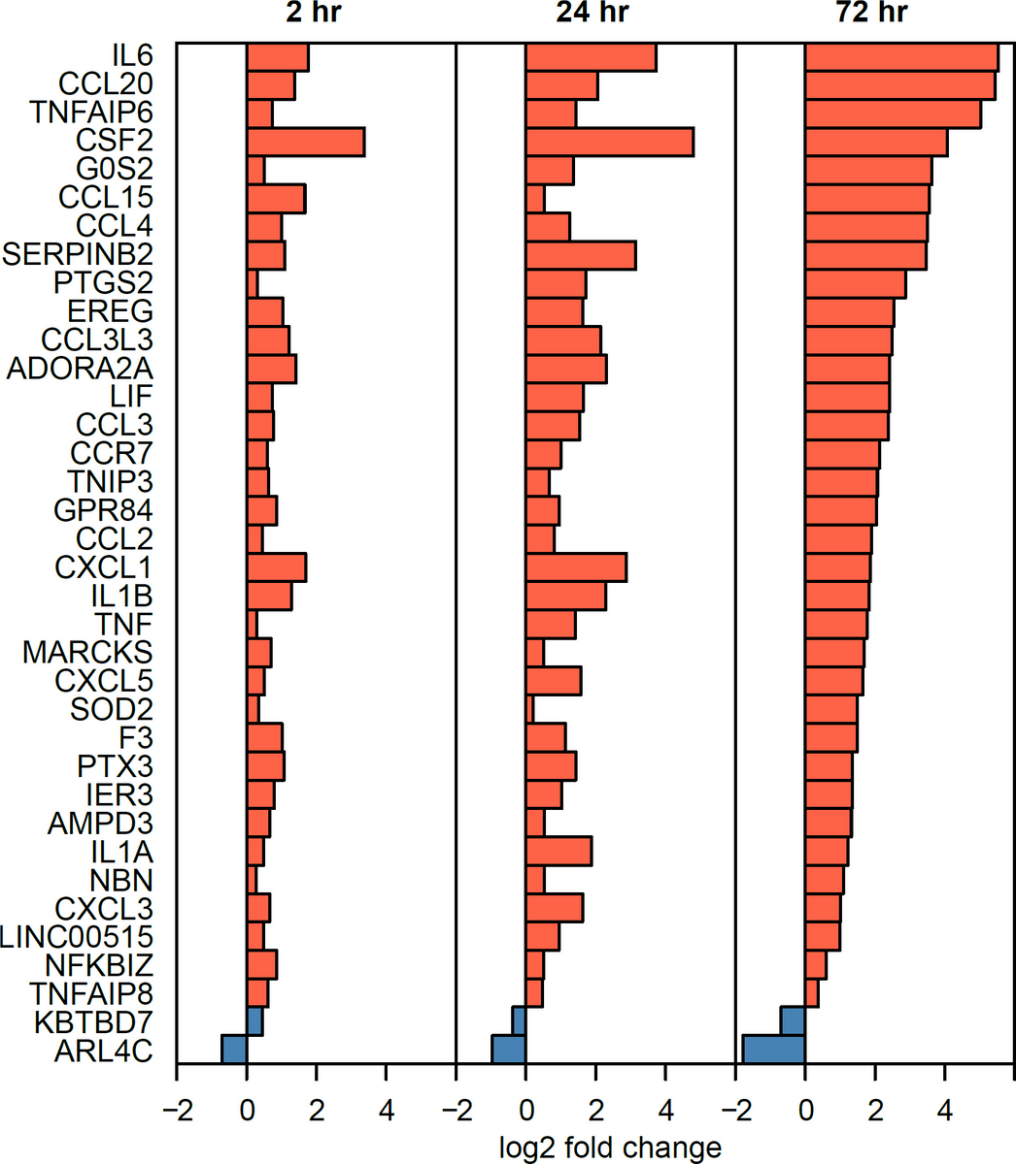
The transcriptional response of 28 human AMs to *M.tb* infection. Shown are the 36 significant DE genes common to all time points (see Figure 3A), and their average log_2_ fold change.

To identify significant DE genes (FDR adjusted p ≤ 0.05) specific to infection, we analyzed RNA expression by comparing uninfected control cells to infected cells at each time point (30). This yielded 62 DE genes at 2h, 2,177 at 24h, and 3,662 at 72h post infection (**Additional file 6: Table S4**). In a previous study also using AmpliSeq (30) on macrophages from three donors, we had detected 16 DE genes at 2h, 899 at 24h, and 174 at 72h, showing substantial overlap with DE genes in this larger study. Thirty-six DE genes were significant at all-time points (Fig. 3A and Fig. 4), including those encoding inflammatory cytokines (IL1A, IL1B, TNF) and chemokine receptors (*e.g*., CCR7) characteristic of a spectrum representing “M1 type” cell states.

Our cell type deconvolution identified changes in the abundance of M1-like and M2-like macrophages as a major difference between control and infected cells. As deconvolution is contingent on the reference data sets we sought to further investigate the relationship to M1/M2-like macrophages and differential expression. Using an independent assignment of M1- and M2-associated genes (31) we confirmed a high overlap of DE genes at each time point with either M1 or M2 genes (Fig. 3B–E), accounting for 44.0% of M1 genes and 34.0% of M2 DE genes at 72h. While individual donors varied significantly in the expression of several characteristic M1 or M2 genes, significant correlations were not detectable between M1/M2 profiles and *M.tb* growth dynamics. While our data support the relevance of ‘M1’-like states to *M.tb* infection, polarization into either ‘M1’ or ‘M2’-like states is increasingly recognized as a false dichotomy with macrophages residing across a spectrum of states. For example, AMs express both M1 and M2-like gene signatures (32). To assess the states of the macrophages responding to infection we performed gene set enrichment analysis of the 72h DE gene profiles, using gene co-expression modules previously identified from the full spectrum of macrophage differentiation (33). This showed a significant enrichment of M1-like modules (Fig. 3F), supporting our initial observation, but also enrichment of gene modules from other macrophage states.

As one example, induction of the highly significant DE transcript STAT1 (a transcription factor associated with an M1-like state) varied between AM donors from a 2-fold reduction to a 25-fold increase. These results indicate that classically defined M1 and M2 cell types play a major role in the early macrophage responses. Enrichment in gene co-expression modules correlated with a spectrum of macrophage phenotypes [(e.g., modules correlated with TNF, PGE2 and Pam3CysSK4 or P3C (TPP)] stimulation which correspond to chronic inflammation (Fig. 3F). These results are consistent with AMs representing a unique macrophage phenotype that does not fit neatly into a classically defined M1 or M2 cell type but exists along a spectrum of macrophage types (**Additional file 5: Supplementary Fig. 2**) (32, 34–36).

### Differentially expressed genes (DE genes) displaying the most variable expression in AMs between donors (VE genes)

The DE genes we identified capture differences in the transcriptional response between infected and uninfected control cells, although this did not identify genes that show high variability between individuals – one important goal of this study. To capture inter-individual variation in gene expression we performed variance analysis of gene expression in both control and infected AMs at all time-points, yielding a set of 324 genes with highly variable expression (VE genes; Levene’s test, ratios of variances < 0.15; **Additional file 7: Table S5A**). All 324 VE genes are also significant DE genes which were found to be upregulated in and specific to the infected AMs. Examples include *IFI6, IL1B, CCL4, IDO1, GBP5, IRF1, JAK3, UBD, CXCL5, CCL20, VDR, CD80, IFI44L, NLRP3*, and *IL7R*, several of which were previously implicated in TB pathogenesis (37). Correlations between expression of these genes with *M.tb* generation times are listed in **Additional file 7: Table S5A.**

**Figure 5:**
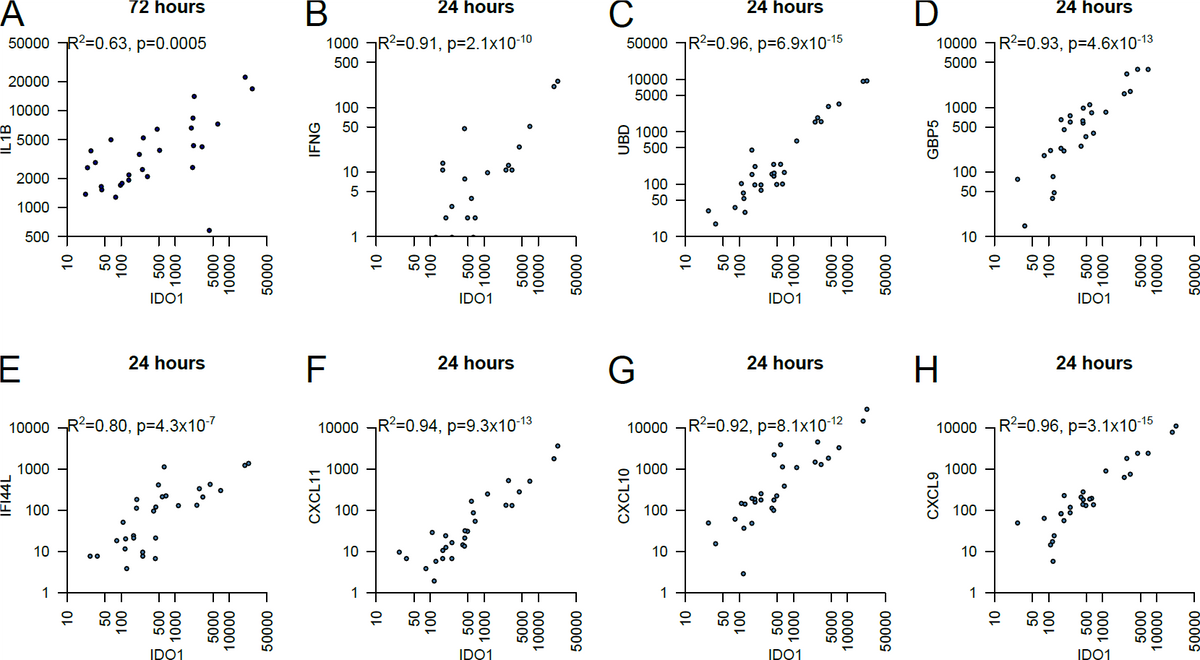
Expression of *IDO1* compared to *IL1B* and other co-expressed genes in AMs infected with *M.tb* at 24 or 72h. (**A**) Expression of *IDO1* and *IL1B* at 72h: both are variably expressed with a different pattern across the 28 AMs, across samples the expression of *IDO1* and *IL1B* is correlated. (**B-H**) Select mRNA transcripts with co-expression patterns similar to *IDO1* at 24h. *IDO1* is poorly expressed in 18 of 28 AMs (mapped fragments per million reads <500) (**A**), with tightly co-expressed genes representing the most variably expressed gene cluster (**B-H**).

Among the VE genes, expression of *IDO1* (a well characterized marker of *M.tb* infection) was substantially stimulated by *M.tb* in only 10 of the 28 donor AMs (Fig. 5A). *IL1B* was more broadly expressed, but with high expression mostly coinciding with high *IDO1* expression (Fig. 5A), similar to several other key genes (*e.g*., *IFNG, UBD, GBP5, IFI44L, CXCL9–11*; Fig. 5B–H). These most variably expressed VE genes, including *STAT1*, are likely relevant to inter-individual variability following infection of AMs. A degree of co-expression of genes with proposed opposing effects on *M.tb*, e.g., *IL1B* and *IFNG* (better control) versus *IDO1* and *Type 1 IFNs* (reduced control) – suggests counteracting influence on growth during the early phase of infection and requires further experimentation.

**Figure 6:**
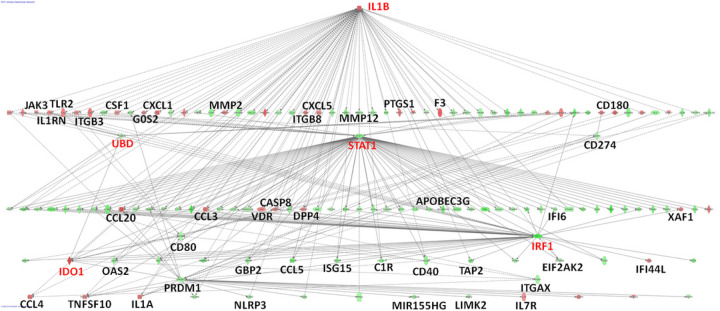
Network of DE genes with highly variable RNA expression (VE/DE genes). These VE/DE genes (n=324) were identified in the 28 AMs after *M.tb* infection across all time points. Standard pathway enrichment program, Ingenuity Pathway Analysis (IPA) (https://www.qiagenbioinformatics.com/products/ingenuity-pathway-analysis/), generates a top scoring gene network with *IL1B*, *UBD*, *STAT1*, and *IRF1* as key hub genes. *STAT1* and *IRF1* cooperatively bind to the promoter of *IDO1*, which is also highlighted (fully annotated network is depicted in Additional file 8: Supplementary Fig. 3). These key gene names are highlighted in red.

Reactome analysis yielded several significant pathways, including Immunological Diseases/Cellular Function and Maintenance/Inflammatory Response, and Function/Immune Cell Trafficking (**Additional file 7: Table S5B**). Ingenuity Pathway Analysis (IPA) of the VE genes yielded the highest scoring hierarchical network with IL1B at the top, and with STAT1, IRF1, and IDO1 as connected nodes (Fig. 6, full gene annotation in **Additional file 8: Supplementary Fig. 3**). Both *STAT1* and *IRF1* interact with the *IDO1* promoter (38) and play a role in macrophage polarization.

### Identification of human AM gene networks associated with M.tb growth

Single RNA profiles across the donor AM dataset displayed high correlations with *M.tb* growth dynamics but failed to yield significance upon FDR corrections. For future studies, it is likely that increased sample size will be valuable in the identification of correlations between growth and single gene expression. Several candidate genes strongly correlated between reads per millions (RPMs) at 24h and generation time, suggesting biologically relevant relationships. As naïve FDR approaches do not account for the correlation between the expression of groups of genes a common approach is to collapse the expression of genes which show highly similar patterns into gene expression modules, with each module randomly assigned a color for identification. Eigengenes representing the expression level of genes within a module for an individual can then be correlated with a phenotype. To this end we built three consensus networks using batch-corrected RNA profiles from infected and control cells at 2, 24 and 72h, with RNAs having RPMs > 35 using WGCNA. This partitioned 9,193, 9,098 and 9,115 genes into 26, 27, and 37 modules for the 2, 24 and 72h time points, respectively (**Additional file 9: Table S6A**). Correlation between network eigengene modules for infected cells and *M.tb* generation times (during 48–72h) at 2, 24 and 72h identified four significant associations between bacterial growth rate and eigengene modules, all in the 24h network (FDR-corrected p-values < 0.05; blue r=−0.52, yellow r=−0.61, red r=−0.55 and tan r = 0.52; **Additional file 10: Table S7**, columns D,E, modules highlighted in red). Reactome pathway over-enrichment analysis (**Additional file 11: Table S8**) showed Processing and Metabolism of RNA, and DNA Damage/Repair as significantly associated with these gene modules. Regulation of genes involved in DNA repair and recombination had been linked to *M*.*tb* regrowth from its non-replicating, persistent state (39). This result supports a nexus between gene networks and *M.tb* generation times, supporting the notion that *M.tb*-stimulated AM gene expression patterns modulate bacterial growth that vary between individuals.

To prioritize individual genes within significantly associated modules, we measured correlations between generation time and gene expression for each gene within the four significant modules (**Additional file 9: Table S6B**). We lowered the stringency of our approach by performing multiple test correction based upon the module size, rather than for all expressed genes. This approach yielded 143 significant genes, with 139 genes belonging to the yellow module in the 24h network (97.2%). The yellow module genes had mostly negative correlations with generation times (117 of 139 genes, 84.2%); *i*.*e*., elevated expression of these genes was associated with shorter generation times (higher growth rates), perhaps signaling a key *M.tb*-induced AM response favoring bacterial growth.

**Figure 7:**
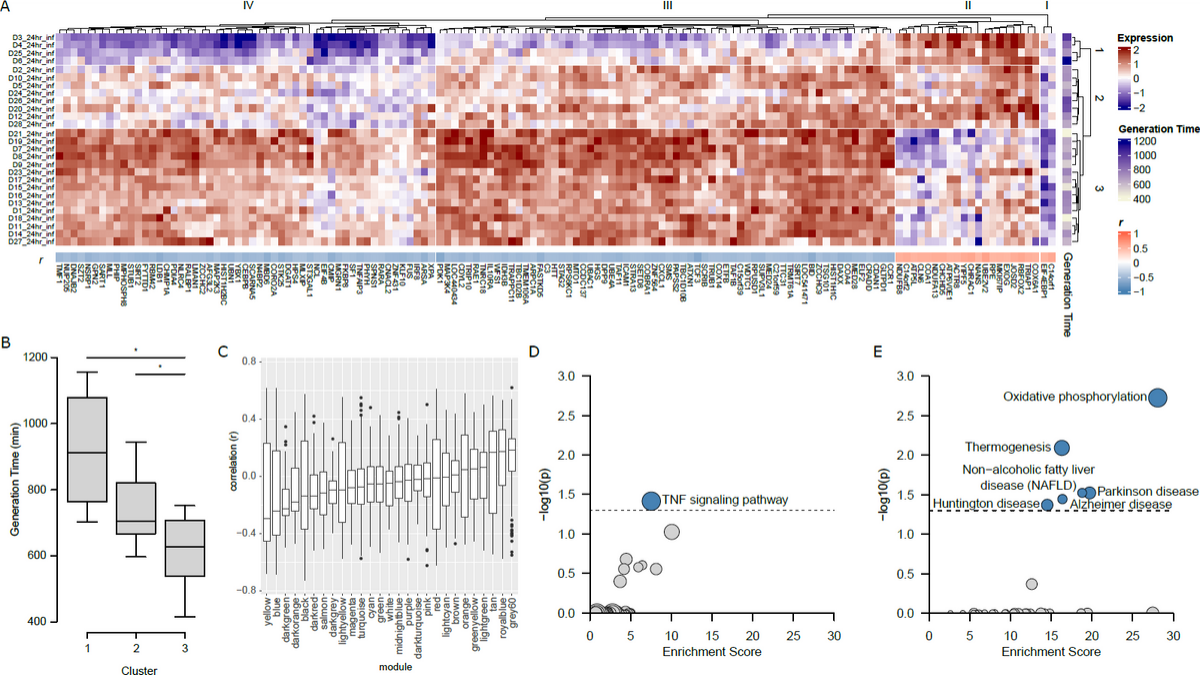
Association of the yellow eigengene module from 24h samples with generation time. (**A**) Gene expression profile of 139 genes from the yellow eigengene module identified by WGCNA which are correlated with generation time. Each gene is Z-normalized across all samples for ease of presentation (Expression). To the right of the heatmap is the generation time in minutes for each sample (Generation Time), and a dendrogram capturing the distance between samples. Below the heatmap are the r values for gene expression against generation time. Above the heatmap is a dendrogram capturing the distance between gene expression profiles. Three clusters of individuals were identified from the gene expression data (1–3). (**B**) The boxplot displays the generation time for individuals within each participant cluster; both cluster 1 and cluster 2 have significantly higher generation times than cluster 3. (**C**) The boxplot shows the distribution of r^2^ values for all modules from 24h expression data. (**D**) KEGG pathway enrichment for negatively correlated genes (clusters III and IV). (**E**) KEGG pathway enrichment for positively correlated genes (clusters I and II).

We performed hierarchical clustering of gene expression profiles and AM donors for genes within the yellow module from the 24h network, limiting to genes where we found a significant correlation between generation time and expression level (Fig. 7A). This revealed four groups of genes, two of which contained genes with a positive correlation to generation time (clusters I and II), and two with a negative correlation to generation time (clusters III and IV). Hierarchical clustering also identified three groups of AM donors, with groups 1 and 2 displaying significantly longer generation times compared to group 3 (Fig. 7B). The correlation coefficients (r^2^ values) were also determined from 24h gene expression data to assess their distribution across all modules (Fig. 7C).

Gene expression sub-groups displayed opposite profiles between groups 1 and 3, with group 2 in between. We performed KEGG pathway enrichment of gene expression clusters I and II (positively correlated with generation time) and clusters III and IV (negatively correlated with generation time). This identified the TNF signaling pathway as significantly enriched in negatively correlated genes (Fig. 7D), and oxidative phosphorylation and thermogenesis significantly enriched in positively correlated genes (Fig. 7E), both were unexpected results. The co-expressed gene group representing a TNF signaling pathway with rapid growth does not include TNF itself, and is likely a consequence of rapid *M.tb* growth. The slower *M.tb* growth rate in group 1 is likely reflective of less *M.tb*-mediated inhibition of oxidative phosphorylation. Only some genes involved in oxidative phosphorylation genes fell into group 3, suggesting overlapping regulatory pathways with differential effects on *M.tb* growth. Nevertheless, our results further support a biological relationship between gene expression profiles and *M.tb* generation times.

### Eigengene modules and the secreted protein response to M.tb infection in human AMs

Among the measured proteins implicated in TB pathogenesis, IL10 and MMP10 were significantly correlated with bacterial growth (Fig. 1). Although in our *in vitro* system the other measured proteins did not show an association with *M.tb* growth, they are all known to impact TB disease progression through interactions with the immune system and the regulation of their expression is of major interest (40). To identify the transcriptional modules that may regulate or respond to these proteins, we assessed correlations between eigengene modules and secreted protein levels (**Additional file 12: Table S9**), identifying 29 significant correlations after Bonferroni correction. We opted for Bonferroni correction at this point for greater stringency in capturing correlations. Six of twenty-nine significant correlations were in control AMs, 3 of which were at the 2h time point where there was little divergence in gene expression between control and infected conditions. Of these, CCL3 expression was strongly correlated with the royal blue module [**Additional file 11: Table S8** (column b) **and Additional file 12: Table S9**] and may be driven by *ex vivo* adaptation of macrophages.

In the 24h network, we found three modules with a significant link to *M.tb* growth rate and to both host transcriptional and protein profiles (**Additional file 9: Table S6B and Additional file 10: Table S7**). These are CCL3 (blue, red and yellow modules) and IL10 (red module). Significant correlations between these gene modules and individual proteins also suggest a causative relationship between gene expression and *M.tb* growth. At 72h, a single gene expression module (tan) correlated with the expression of multiple proteins (IL15, IL18, IL1A and TNF). This module overlapped substantially with our highest scoring network from IPA analysis of variably expressed VE genes (**Additional file 8: Supplementary Fig. 3**) with 47 of 184 module eigengenes (25.4%) falling within this network, including *IDO1*, *IL1A* and *IL1B*.

#### Replication study of freshly isolated AMs in a different population using RNA-Seq

The goal of this part of the study was to test whether application of full RNA sequencing in AM samples from a different population in South Africa provides an overlapping set of DE genes in AM samples of our current study population. This population included 11 donors used as healthy controls with QuantiFERON positive status (Table 1 cohort A). *M.tb* H_37_R_v_ was the infecting strain. DE genes are presented in **Additional file 13: Table S10** and **Additional file 14: Supplementary Fig. 4**. At 2h post infection, fewer RNAs were assigned DE status (45), and only 3 upregulated RNAs overlapped with the DE genes displayed above, likely owing to the small number of samples and the less precise and less targeted RNA-Seq methodology. However, at 24h, 360 of 773 DE genes (46.6%), and at 72h, 89 of 148 DE genes (60.1%) overlapped with the DE genes reported in the main study, although the donors of two different demographic cohorts were genetically very diverse and of different ages. Pathway analyses revealed a similar spectrum of enriched functional terms for upregulated genes, including cytokine signaling in immune system, interferon signaling, and innate immune system. Inspection of key DE genes, irrespective of AM donors and infection time points, replicates the finding of substantial inter-individual differences, for example *IDO1* and its co-expressed genes (**Additional file 15: Supplementary Fig. 5**).

## Discussion

The protein and RNA expression profiles parallel large inter-individual variation in interactions between healthy human AMs and a single virulent strain of *M.tb* (Figs. 1 and 2, measured with RLUs). For the first time this study dissects biological mechanisms underlying individual differences. Sousa et al. (9) had demonstrated that secretion of IL1B is a surrogate marker distinguishing between TB cases with mild disease and severe TB, attributing differences in IL1B induction to virulence of the strains tested, independent of the host. However, here we show that a single virulent *M.tb* strain elicits substantial differences in IL1B expression between individual AMs, highlighting host factors, with IL1B one possible predictive marker of TB susceptibility. A smaller replication study identifies an overlapping set of DE genes and confirms substantial inter-individual variability of critical DE genes such as *IDO1* (Additional file 15: Supplementary Fig. 5).

The nexus between *M.tb* growth rates and secreted proteins and eigengene modules further supports the hypothesis that gene expression networks, both in uninfected and *M.tb*-infected cells, affect *M.tb*-AM interactions. While we have applied stringent FDR adjustments to reveal significant correlations, strong candidate genes and networks can serve to extract a deeper understanding of the various phases of *M.tb*-AM interactions and their possible significance in the pathogenesis of TB.

We first measured 27 candidate proteins that are secreted by *M.tb*-infected AMs and uninfected controls, finding robust stimulation by *M.tb* and inter-subject differences (Fig. 1C–H). Importantly, two proteins (IL10 and MMP10) are significantly correlated with bacterial growth over 48–72h (Additional file 3: Table S3), suggesting the hypothesis that proteins relevant to TB pathogenesis affect early *M.tb*-AM interactions, possibly presaging individual susceptibility to TB. Production of both IL10 and MMP10 have been reported to promote the disease caused by another bacterial pathogen, e.g., *Helicobacter pylori* (41, 42).

RNA expression profiles also appear to reflect or influence *M.tb*-AM interactions. The precision of the AmpliSeq RNA assay, together with use of control uninfected AM expression at each time point (30), enabled sensitive detection of numerous DE RNAs across the 28 donors for AMs (Fig. 3; Additional file 5: Table S4). DE genes significant at each time point (Fig. 4) are consistent with previously reported results [*e.g*., (22, 28, 30)], including inflammatory cytokines CSF2, IL6, IL1B, and IFNG, but also anti-inflammatory factors such as CCL22. Gene Ontogeny (GO) Pathway analysis reveals the expected prevalence for Innate Immune System, Cytokine Signaling in Immune System, and more.

Macrophages were initially characterized as polar phenotypes, i.e., M1 or M2 (43). However, AMs represent a unique spectrum of phenotypes and express M1 and M2 markers (Additional file 5: Supplementary Fig. 2) (35). In this study, we used published modules (31) that have defined classical M1 or M2 type genes as the starting point. We found that the DE genes include both a classically activated M1 type induced by inflammatory agents (*e.g*., IFNG) and alternatively activated M2 type induced by IL4 and IL13 (20). The M1 type is strongly enriched in infected samples, while M2 markers increase at 72h particularly in the uninfected controls (Fig. 2; Additional file 5: Supplementary Fig. 2). Substantial differences between M1 and M2 markers occur between individuals, reinforcing variability of an individual’s response to infection. The balance of this mixed mode of activation may account in part for inter-subject differences in *M.tb*-AM interactions, but no significant trends were observed with respect to growth rates. In previous reports, *M.tb* ESAT-6-induced macrophage polarization to M1 phenotype occurred early, then switched to M2 phenotype at a later stage of infection (44). In our study, a switch to the M2 phenotype at a later stage (72h) was also observed in uninfected control macrophages compared to infected AMs, which could be due to increased expression of MMPs in control cultures. Upregulation of certain MMPs was found to be associated with macrophage polarization to both M1 and M2 phenotypes in *M.tb*-infected, cigarette smoke-exposed macrophages (45). The majority of individuals in our primary cohort were non-smokers. Future work on the impact of smoking and MMPs can be best assessed in the African replication cohort which were predominantly smokers.

Among the gene co-expression modules, one (yellow) significantly correlates with *M.tb* generation times and, in addition, contains numerous individual genes that also significantly correlate with *M.tb* growth (Additional file 9: Table S6). As most correlations are negative, these genes appear to favor *M.tb* growth. The module GO terms indicate influence over translation processes, a potential mechanism by which *M.tb* usurps the cellular machinery to its advantage (46).

VE genes can reveal markers of individual susceptibility or resistance to disease phenotypes (47), tested here for *M.tb* infection of AMs. We find that all of the most robust VE genes also classify as DE genes, likely contributing to the fate of *M.tb* in the cells. Prominent examples include VE/DE genes that encode IL1B and IDO1 (Additional file 7: Table S5), suggesting that VE/DE genes are relevant to infection and disease progression. IPA analysis of VE/DE genes yields Interferon Signaling as the top pathway, and a network with IL1B, STAT1, and IRF1 as dominant hubs including genes previously implicated in the cellular response to *M.tb* [e.g.,(21, 22, 28)] (Additional file 8: Supplementary Fig. 3). This IL1B-dominated network connects IL1B, STAT1, and IRF1 to IDO1 (Fig. 6), consistent with previous reports that the IRF1/STAT1 transcription complex binds to the *IDO1* promoter (38, 48).

We observed a significant increase in GBP2 expression in *M.tb*-infected macrophages versus controls at 24 and 72h (highly significant at 72h). GBP2 is one of the genes in the blood based RISK6 transcriptome proposed as a biomarker for TB disease and treatment response (49). However, GBP2 expression did not correlate with *M.tb* growth generation times (Additional file 7: Table S5A). Expression of the other 5 genes in the RISK6 signature was not significantly different in *M.tb*-infected AMs, suggesting some specificity for blood *vs* tissue responses. IDO1 has been proposed recently as a sensitive and selective biomarker for active TB (26). Its metabolic products, immunosuppressive kynurenins, act by preventing access of cytotoxic T cells to infected macrophages in TB lung granulomas (27). IDO1 is robustly expressed in only 10 of 28 AMs after *M.tb* stimulation, with a correlated expression profile for IL1B, CXCL9–11, IFNG, UBD, IFI44L and GBP5 (an IFN/IL1B activated GTPase mediating antibacterial defense) (Fig. 5). We propose that *M.tb*-induced expression of both IL1B and IDO1 could be early predictive indicators of susceptibility to *M.tb* for a given individual if similar results are obtained in more readily accessible cells (*e.g*., MDMs). Indeed, increased macrophage IL1B expression was observed in non-human primate granulomas that are more permissive to *M.tb* growth (50). However, increased IL1B could simply be increased secondary to bacterial burden. Increased IL1B activity is typically associated with *M.tb* control (51).

The nexus we observe between secreted proteins reported relevant to TB and gene expression modules, with overlap to variably expressed VE/DE genes and, on the other hand, to *M.tb* generation times, strengthens our hypothesis that early *M.tb*-AM interactions could signal an individual’s susceptibility to infection and potentially risk of overt TB.

## Conclusions

In summary, our results identify known and novel key genes and their encoded transcripts and proteins in the early phase of *M.tb* infection of freshly isolated human AMs. Previous work shows that infection of macrophages with *M.tb* stimulates multiple pathways, including both interferon type I and II pathways – thought to exert opposing effects on infections (20, 21). We show here that variably and differentially expressed genes and their networks affecting *M.tb*-AM interactions are associated with inter-individual variability during the early phase of infection. Results of this study provide a foundation for identifying predictive biological indicators of an individual’s susceptibility or resistance to *M.tb* infection, and response to therapies and vaccines (52).

## Limitations

The current study employs freshly isolated AMs (used within 6h of harvest) and hence is distinct from MDMs isolated from peripheral blood after 5 days of *in vitro* incubation, accounting for substantial differences of RNA profiles between them (30). However, during AM incubation for 72h, we observed substantial transcriptome changes even in the absence of infection, with a tendency towards the profile of MDM transcriptomes as we have found previously (30). Nevertheless, the cultured AMs maintain distinct characteristics from MDMs over the incubation period. We have mitigated any *in vitro* artifacts with use of AM controls at each time point for each donor. In addition, very large inter-individual differences occur at 2h post infection, likely caused by variable cellular adaptation of the *M.tb*-lux strain and thus we opted not to focus on this time period but can pursue further in the future. While the study group of 28 healthy AM donors was sufficient to reveal significant genes associated with *M.tb*-AM interactions (this is a relatively large study given the difficulty of obtaining fresh AMs from healthy individuals), supported by a smaller replication cohort, functional studies will be required to establish causal relationships. Also, larger cohorts will be needed to address contributions of single genes and networks to uptake, adaptation, and growth, reveal genetic factors, and assess ethnicity, sex and age, and environmental factors. Nevertheless, the study approach provides rich datasets to aid in facilitating development of predictive biomarker panels of an individual’s risk for TB. The design of our study precluded repeat measures from each donor. However, our longstanding studies with healthy donor human MDMs has demonstrated that individual donors provide consistent results for comparable measurements over time.

## Methods

### Measurements of uptake, adaptation, and growth rate of M.tb in infected AMs

Fresh AMs were obtained, prepared and cultured within 6h from 28 tuberculin skin test (TST)-negative healthy donors from Caucasians, Asians, and Africans, according to the demographics of the Columbus, Ohio area (Table 1), under an approved IRB protocol at the Ohio State University Wexner Medical Center. Isolation and culture of human AMs from bronchoalveolar lavage (BAL) was done as described (53, 54). Briefly, BAL fluid was centrifuged and washed once in cold RPMI at 4°C, and the cell pellet was resuspended in RPMI medium. A portion of the cell suspension was subjected to cytospin followed by staining and microscopy to determine macrophage content (94 ± 5%; mean ± SD, N = 10). AMs were adhered for 2h in either a 24-well plate (1.5×10^5^ cells/well) or 96-well plate (5×10^4^ cells/well) in RPMI containing 10% human AB serum and Penicillin G (10,000 U/ml) (~ 99% pure). The resultant uninfected HAM monolayers were then washed, and cultured in RHH media for another 2h (this is the time period where *M.tb* was added to separate wells for the infection). Supernatants were collected for protein and cell monolayers lysed for RNA analysis. For longer time points, monolayers were washed, medium replaced, and incubations continued for 24 or 72h for repeat protein and RNA analysis.

For *M.tb* infection of HAMs, virulent *M.tb* H_37_R_v_ single cell suspensions were prepared (55). The virulent *M.tb* strain used contains the entire bacterial Lux operon cloned in a mycobacterial integrative expression vector (*M.tb* H_37_R_v_-Lux) as described (29). *M.tb* cellular uptake, adaptation, and intracellular growth were assessed as relative luminescence units (RLUs) at 2, 24, 48, and 72h in 3–5 wells for each condition (mean values of 3–5 technical replicates are provided for each donor) using a multiwell plate reader (Glomax, Promega). Each 2h incubation was performed at a multiplicity of infection (MOI) of both 2:1 and 10:1 (*M.tb*/AM cells for RLU assays); RNA and protein was measured only with MOI of 2:1.

For technical reasons, AM uptake and growth assays from donors 1–16 were incubated in 24 well plates, while AMs from donors 17–28 were incubated in 96 well plates. Use of the different plates resulted in ~ 3-fold difference in mean RLUs between the two sets of donor groups (directly related to a different number of AMs/well). For all analysis we performed batch correction of protein, transcript and growth rate measures using ComBAT (56) to remove the impact of different plates used during culture as such we specified the batches as individuals 1–16 and 17–28.

### Measurement of secreted proteins in control and M.tb -infected AMs

Supernatants from culture wells of uninfected and infected AMs from 28 donors at 2, 24, and 72h were analyzed for secreted proteins relevant to *M.tb*-macrophage interactions using multiplex kits from Meso Scale Discovery (MSD) (Rockville, MD). The supernatants were collected at 2h and replaced with fresh medium, followed by continuing incubation until 24 or 72h. Twenty-seven secreted proteins were measured: 4 inflammatory mediators (TNF-α, IL-6, IL-1β, IL-10; V-PLEX kit), 6 cytokines [CSF2 (GM-CSF), IL-15, IL-16, IL-1α, IL-7, VEGFA] and 12 chemokines [IP-10 (CXCL10), MCP-1 (CCL2), MCP-4 (CCL13), MDC (CCL22), IL-8, TARC (CCL17), MIP-1α (CCL3; U-PLEX (customized multiplexing)), MIP-1β (CCL4), ENA-78 (CXCL5), IL-18, MIP-3α (CCL20), MIP-3β (CCL19)], and 5 matrix metallo-proteinases (MMP-1, MMP-2, MMP-3, MMP-9, MMP-10; MMP 3-PLEX and MMP 2-PLEX).

### Measurement of RNA expression from uninfected and M.tb -infected human AMs

Expression of 20,804 RNAs, including 2,228 non-coding RNAs (ncRNAs), was measured with AmpliSeqTM (Whole transcriptome Human Gene Expression Kit, Life Technologies) for 28 donor AMs at 2, 24, and 72h after infection, for both uninfected controls and infected AMs at each time point (MOI 2:1). AmpliSeq transcriptome analysis incorporates a targeted, amplicon-based (~ 110 bps, spanning exons) workflow, and is quantitative over orders of magnitude. The precision of AmpliSeq analysis detects *M.tb*-induced expression changes with high sensitivity in human MDMs and AMs infected with *M.tb* (30). Genomic DNA and total RNA (TRIzol^®^ Reagent (Ambion^™^, Austin, TX)) were prepared from AMs using published procedures (11). RNA was purified, DNase-treated, RNA concentration measured, and RNA integrity assessed as described (30). After reverse transcription of 10 ng total RNA, using the AmpliSeq primers with the SuperScript^®^ VILO^™^ cDNA Synthesis kit the cDNAs were amplified for 12 cycles with Ion AmpliSeq^™^ primers and barcoded adapters, resulting libraries purified and pooled in equal amounts for emulsion PCR on an Ion OneTouch^™^ 2 instrument, followed by sequencing with the Ion Proton^™^ sequencer (30). Reads were aligned to BED (Browser Extensible Data) file specific for AmpliSeq amplicons. Typically, we obtain 5–9 million mappable reads per sample, with ~ 50–60% of RNA targets detected (30). Repeat experiments in the same sample yield correlation values of r^2^ > 0.99 (for both independent replicates and sequencing chip replicates). The AM sample from D17 at 2h and 72h yielded < 1 million reads and were excluded from the analyses. The AmpliSeq reads were normalized to mapped fragments per million reads for quantifying transcript expression levels (57), yielding relative abundance for predicted transcripts in each AM.

### Differentially-expressed (DE) genes between control and M.tb -infected human AMs

To identify DE genes, we employed DESeq2 (58). FDR adjusted p values of 0.05 were used as a cutoff for identifying DE genes at each time point. We estimated size factors using the “poscount” approach to correct for different sequencing depth and performed independent analysis at each time point, specifying the individual/donor and condition (infected or control) in the model.

#### Weighted gene co-expression network analysis) (WGCNA)

We derived three gene co-expression works using WGCNA (59) with the following parameters; power = 8, TOMtype = signed, minModuleSize = 30, mergeCutHeight = 0.25, minKMEtoStay = 0. These were blockwise consensus modules with the gene expression data from the control and infected cells grouped independently. We correlated the module eigengenes to either bacterial growth rate, or to proteins expressed/secreted from the same time point and performed FDR correction on p values.

### Detection of genes with variable expression (VE) in control and M.tb -infected AMs, characterized by variance measures

To identify the most variably expressed (VE) RNAs in control AMs and in those separately after *M.tb* infection, all AM transcriptome data at each time were subjected to Levene’s test (60), with ratios of variances as test statistics, reported adjusted p-value (FDR) for selected RNAs. A second variability test assesses whether the entropy of a given RNA’s expression is higher than expected given the total entropy in the *M.tb*-treated AMs (61). A permutation test yields p-values for significance of the entropy computations.

#### Gene pathway and ontogeny analysis

We performed over representation analysis of gene ontology (GO) terms and reactome pathways using WebGestalt (62) taking the relevant gene lists for each comparison as an input and using default parameters. We retained GO terms or pathways which reached an FDR corrected p value of 0.05. Comparison to genes associated with M1 and M2 states was performed using a curated list of genes from Viola et al. (63) and Li et al. (64).

##### Replication study of AMs in South Africa

AMs were collected from 11 close contacts of TB patients (n = 11) in Cape Town, South Africa (Table 2), under the approval of the Health Research Ethics Committee of Stellenbosch University. Close contacts were defined as individuals who shared a closed space with a newly diagnosed TB patient for at least 5 hours per week (all contacts were QuantiFERON positive). These TB household close contacts were used as healthy controls. The processing of BAL fluid to isolate AMs and their downstream uses were performed as described above for the main cohort from Columbus, Ohio with minor variations. AMs were adhered and cultured in 96-well plate (1.3×10^5^ cells/well, in triplicate) in RPMI medium containing 20% AB serum. Cells were infected with wild-type *M.tb* H_37_R_v_ at an MOI of 1:1. CFU, RNA and protein were measured at 2, 24 and 72h.

AM samples were initially stored in RNAlater and transferred to TRIzol prior to shipment. RNA was isolated using Quick-RNA Microprep Kit (R1050; Zymo Research) as per the manufacturer’s instructions. Isolated RNA was quantified using the Qubit RNA High Sensitivity Assay Kit (Q32852) and Qubit 4 Fluorometer (both from ThermoFisher Scientific). Before doing RNA sequencing, RNA quality was assessed with the Fragment analyzer (Agilent). Samples with an RNA integrity number (RIN) higher than 7 were used for RNA-seq. RNA-seq libraries were prepared from 25–500ng of Total RNA using the NEB Next directional RNA library preparation Kit with poly (A) enrichment module (Ipswich, MA). RNA sequencing was carried out at the Genome Sequencing Facility (GSF) at UT Health San Antonio using the HiSeq 3000 platform (Illumina), with 50bp single read sequencing with approximately 25M reads per sample.

The analyses of RNA-Seq data of the South Africa cohort samples were carried out using CLC Genomics Workbench 22 (65). Low-quality sequences and adapters were trimmed. Trimmed reads were mapped to the human hg38 reference genome. The reads were assigned to the transcripts using the EM algorithm. The TMM normalization (66) in EdgeR (67, 68) and multi-factorial statistics based on a negative binomial Generalized Linear Model (GLM) were carried out for differential expression analysis. Genes with FDR- adjusted p-values < 0.05 were considered to be statistically significant. Gene Set Enrichment Analysis (GSEA) (69) and KOBAS analysis (70) were carried out to identify over-represented gene ontology (GO) terms and biological pathways.

## Availability of data and materials

The RNA sequencing data were deposited into the GEO database under accession numbers GSE189996 and GSE223863.

## Figures and Tables

**Table 1:** Donor demographics of AM samples used in this study (n = 28)

Donor	Sex	Age (Years)	Race	Donor	Sex	Age (Years)	Race
**D1**	Female	24	Asian (Chinese)	**D15**	Male	18	Hispanic
**D2**	Male	24	African-American	**D16**	Female	25	Caucasian
**D3**	Male	27	Caucasian	**D17**	Female	20	Caucasian
**D4**	Female	26	Caucasian	**D18**	Female	26	Caucasian
**D5**	Female	26	Caucasian	**D19**	Male	19	African-American
**D6**	Male	21	Caucasian	**D20**	Female	22	Hispanic
**D7**	Male	24	Caucasian	**D21**	Male	20	Asian (Chinese)
**D8**	Female	22	Caucasian	**D22**	Female	19	Not stated
**D9**	Male	23	Caucasian	**D23**	Female	19	Caucasian
**D10**	Male	21	Caucasian	**D24**	Male	23	Caucasian
**D11**	Female	46	Caucasian	**D25**	Male	24	Hispanic
**D12**	Female	22	African-American	**D26**	Male	23	Caucasian
**D13**	Male	27	Asian (Indian)	**D27**	Female	20	Caucasian
**D14**	Male	33	Asian (Chinese)	**D28**	Female	19	Caucasian

Male/Female = 14/14; Caucasian = 60% (17/28), Asian = 14% (4/28), African-American = 11% (3/28), Hispanic = 11% (3/28)

**Table 2:** Healthy control demographics of AM samples obtained from South Africa (n = 11)

*Donor ID	Sex	Age (Years)	**Race
**A257** (LS_109–120)	Female	62	SAC
**A258** (LS_121–126)	Male	51	SAC
**A297**	Female	57	SAC
**A316** (LS_141–148)	Female	46	SAC
**A335** (LS_149–158)	Female	47	SAC
**A349**	Female	57	SAC
**A352** (LS_161–166)	Female	43	SAC
**A367** (LS_169–174)	Female	58	SAC
**A411**	Female	62	SAC
**A415** (LS_177–182)	Female	53	SAC
**A41**6 (LS_183–184)	Female	52	SAC

* The designation in parenthesis refers to a range of RNA sample numbers from the same donor used for RNAseq

** SAC: South African Coloured (mixed ancestry with Khoisan, Bantu, European and Asian roots)
